# Free Light Chains as a Novel Diagnostic Biomarker of Immune System Abnormalities in Multiple Sclerosis and HIV Infection

**DOI:** 10.1155/2019/8382132

**Published:** 2019-12-09

**Authors:** Monika Gudowska-Sawczuk, Barbara Mroczko

**Affiliations:** ^1^Department of Biochemical Diagnostics, Medical University of Bialystok, Waszyngtona 15A St., 15-269 Bialystok, Poland; ^2^Department of Neurodegeneration Diagnostics, Medical University of Bialystok, Waszyngtona 15A St., 15-269 Bialystok, Poland

## Abstract

**Introduction:**

Immunoglobulins are molecules composed of two heavy and two light chains. Light chains are produced by B lymphocytes during the synthesis of immunoglobulins, and physiologically light chains are generally produced in excess compared to heavy chains. Light chains that are not combined to heavy chains in a whole immunoglobulin are called free light chains (FLCs). B-cell abnormalities are associated with disorders leading to an abnormal concentration of free light chains. In this study, we focus on the described changes of serum and cerebrospinal fluid concentration of free light chains in inflammatory disorders: multiple sclerosis, HIV infection, and HIV-associated lymphomas.

**Methods:**

We performed broad research of the literature pertaining to our investigation via the MEDLINE/PubMed database.

**Results:**

It has been proven that FLC determination can provide rapid information about intrathecal inflammation in patients with multiple sclerosis. Moreover, literature data suggest that free light chain determination is the most interesting alternative for oligoclonal band analysis. In the present review, we also described that HIV-related immune system dysfunction is associated with an elevated concentration of serum-free light chains. Additionally, FLCs are potentially a strong and sensitive predictor of the risk of developing HIV-associated lymphomas.

**Conclusion:**

Based on these published findings, we suggest that free light chains have high diagnostic sensitivity, which probably enables application in laboratory diagnostics.

## 1. Introduction

**Figure 1:**
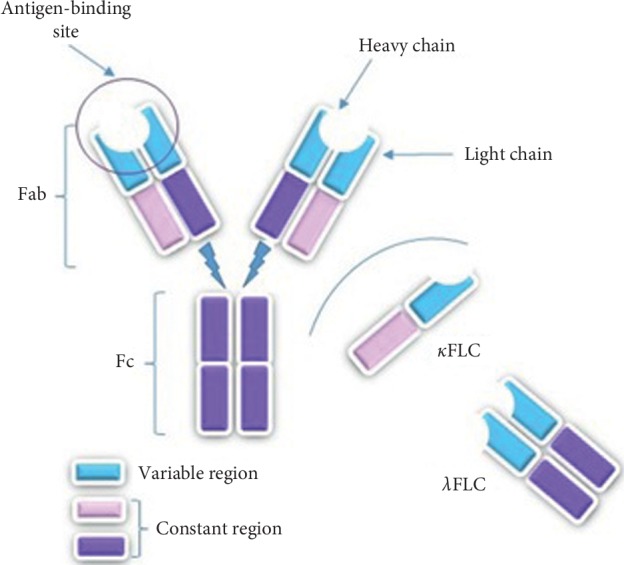


Immunoglobulins are Y-shaped molecules with a tetrameric structure composed of two heavy (H) and two light (L) chains (Figure 1) [1]. Based on the differences of the amino acid sequences in heavy chains, immunoglobulins are divided into 5 classes: G (*γ*), A (*α*), M (*μ*), D (*δ*), and E (*ε*). There are also two types of light chains: kappa (*κ*) and lambda (*λ*), which are covalently linked to the heavy chain. Each heavy and light chain has the constant region and the variable region. One constant and one variable domain of both the chains create the antigen-binding fragment (Fab). The variable domain of Fab contains the paratope that links directly with antigen. Each monomeric antibody has two antigen-binding fragments, so it can bind with two antigens concomitantly. Besides Fab fragment in the structure of an antibody, there is also a fragment called the fragment crystallizable region (Fc region) that interacts with cell surface receptors and activates the immune system [2]. Each immunoglobulin contains always two identical light chains that have length roughly 211–217 amino acids [3–5]. The human gene encoding kappa chain is located on chromosome 2, and the gene encoding lambda chain is located on chromosome 22 [6]. Light chains are produced by B lymphocytes during the synthesis of immunoglobulins, and physiologically, there is always an excess of light chains produced. Light chains that are not combined to heavy chains in a whole immunoglobulin are called free light chains (FLCs). An excess can be around 500 mg/day, but B-cell abnormalities are associated with disorders leading to abnormal concentration of free light chains. Excess free light chains are secreted into the circulation and, usually, light *λ* chains occur as dimeric form and *κ* predominantly as monomeric form [7, 8]. In the circulatory system, FLCs undergo renal clearance which results in a short half-life of 2–4 hours for *λ* and 3–6 hours for *κ* [7, 9].

Early studies showed that serum-free light chain determination represents a routinely usable laboratory marker for the diagnosis of some diseases, including monoclonal gammopathies, e.g., multiple myeloma [10, 11]. However, in this paper, we focus on the described changes of serum- and cerebrospinal fluid- (CSF-) free light chain concentration in other inflammatory diseases: multiple sclerosis (MS), human immunodeficiency virus (HIV) infection, and HIV-associated lymphomas. However, the coexistence of MS and HIV is extremely rare and only a few studies described concomitance of MS and HIV. It has been proven that in patients with HIV infection, neurologic complications remain common and MS is the most common disabling neurological disease in young adults. Moreover, there are some studies suggesting that the MS incidence in HIV-positive patients is lower than that in general population because immunodeficiency induced by this virus may prevent development of MS [12–15]. However, in HIV, the risk of malignancy is significantly increased and more than 40% of HIV-infected people are eventually diagnosed with HIV- or acquired immunodeficiency syndrome- (AIDS-) related lymphoma (ARL) [16].

Indisputably, one common feature of MS and HIV infection is abnormalities of the cellular and humoral immune system. It was described that multiple sclerosis is the only inflammatory disease next to HIV infection, which causes predominant elevation of FLCs in body fluids including CSF, but the reason behind this phenomenon remains unknown [17, 18]. To date, there are only few studies describing the diagnostic usefulness of free light chain measurements in the development of MS and HIV. Thus, we performed thorough research of the literature pertaining to our investigation via the MEDLINE/PubMed database to investigate whether free light chains might be used in the diagnosis of MS, HIV infection, or HIV-related lymphoma, with the aim of improving the prognosis in those patients.

## 2. Multiple Sclerosis

Multiple sclerosis is the most common disease of the central nervous system (CNS) characterized by inflammation and demyelination. The etiology of multiple sclerosis is still unknown. Pathological changes are mainly caused by nerve demyelination which is usually accompanied by axonal deterioration and neuroaxonal loss, mainly affecting the white but also gray matter [19]. A characteristic of this disease is progressive course with multifocal damage of the nervous system [20, 21]. The prognosis of multiple sclerosis depends on the current age of diagnosed patients. Because of that, an early diagnosis is very important. There is no one specific test for the diagnosis of multiple sclerosis, and actually, for diagnostics, the McDonald criteria are used. The last revision of McDonald criteria includes magnetic resonance imaging, symptoms, and CSF examination (the presence of oligoclonal bands (OCBs)) [22].

The diagnostic significance of free light chains in multiple sclerosis has been presented in Table 1. It is well known that in patients with MS and clinically isolated syndrome (CIS), plasma cells present in intrathecal space secrete immunoglobulin G. For the first time, intrathecal immunological stimulation leading to increased synthesis of FLCs within the CNS was observed by Bracco et al. in 1980s [42]. FLC production is an early phenomenon of MS and, for this reason, e.g., Presslauer et al. tried to evaluate the diagnostic utility of free light chain (*κ* and *λ*) measurements in cerebrospinal fluid. They studied samples from over 400 patients where the multiple sclerosis group consisted of patients with relapsing-remitting MS (RRMS) and CIS. They found that in the CSF of the MS patients, the levels of *λ*FLCs were only moderately elevated, but the levels of *κ*FLCs were significantly higher than in other CNS diseases. In contrast, there was no difference between MS and CIS groups. Moreover, in patients with MS, the authors compared these results with OCB and the IgG index calculated according to the following formula: (CSF IgG/serum IgG)/(CSF albumin/serum albumin). In results, they report that *κ*FLCs determination may reflect the intrathecal inflammation. The results correlated with diagnosis of MS and CIS with higher sensitivity than OCB and IgG index, but subtly lower specificity than OCB [17]. Besides *κ*FLCs concentrations, Presslauer et al. tried to evaluate a possible prognostic value of *κ*FLCs index ((CSF *κ*FLCs/serum *κ*FLCs)/(CSF albumin/serum albumin)). They observed that *κ*FLCs index values were significantly higher in MS compared to CIS and *κ*FLCs index values in both and MS and CIS were significantly higher than in the control group [17, 23]. The same results were observed by Voortman et al. and Vecchio et al. in the studies describing the predictive value of FLCs in early multiple sclerosis [24, 28]. Furthermore, Presslauer et al. compared values of *κ*FLCs index in MS and CIS patients with clinical changes over time, but there were no association between *κ*FLCs index and disease progression for the period under review. On the other hand, Voortman et al. observed that the actual levels of *κ*FLCs and *λ*FLCs were not different in regard to disease activity, but the *κ*FLCs index was lower in patients with nonactive disease in comparison to active disease [24]. Presslauer et al. also described that in the OCB positive group, only three patients had *κ*FLCs concentrations within the reference ranges [23]. Similar results were obtained by Menéndez-Valladares et al. who analysed samples from patients with CIS, RRMS, and noninflammatory neurologic diseases. They observed statistically significant differences between CIS and control patients and between RRMS and controls. They also revealed that *κ*FLCs-index has higher sensitivity and specificity than *κ*FLCs concentration what may suggest that the *κ*FLCs concentration is a worse diagnostic biomarker for MS and CIS diagnosis than the *κ*FLCs index. Scientists also highlighted the fact that the risk of conversion of CIS to clinically definite MS correlates with high levels of *κ*FLCs index appointed above in this study cutoff point. The accuracy in prediction of conversion to MS was high for *κ*FLCs index and IgG as well as for OCB independently or together [25]. Valencia-Vera et al. also calculated and compared *κ*FLCs index with other routine tests for intrathecal inflammation and similar to previous studies, they described higher *κ*FLCs index values in MS patients than in patients with other CNS diseases. Second, they showed that *κ*FLCs-index had high sensitivity and specificity for MS, but at cut-off >2.91, specificity was higher and sensitivity was lower than immunoglobulin G oligoclonal band [30]. Moreover, Presslauer et al. observed that some of the patients with MS and CIS with no detectable oligoclonal bands have elevated *κ*FLCs values. It suggests, that *κ* and *λ* free light chains are produced and secreted by cells involved in synthesis of all immunoglobulins, not only IgG [23]. This was also observed by Puthenparampil et al. who showed that in MS, intrathecal IgG synthesis was associated with *κ*FLCs index while intrathecal IgM synthesis correlated with *λ*FLCs index. On the other hand, there was no link between *κ*FLCs index and parameters of intrathecal IgM synthesis. Because of the fact that there was mild correlation between IgM and *λ*FLCs index, the authors emphasize that study needs to be performed in a larger group of patients. Interestingly, in MS patients with no evidence of intrathecal IgG synthesis, they observed the moderate correlation between *κ*FLCs index and *λ*FLCs index, but not in those with the evidence. Consequently, some authors observed lower extend of *λ*FLCs concentration, and therefore, they examined *κ*/*λ* ratio which was increased in MS patients [26, 29]. Interestingly, Rathbone et al. divided cohorts into high and low CSF *κ*/*λ* ratio, observing that there was a significantly lower expanded disability status scale (EDSS) of MS at 5-year follow-up in the group with a high CSF *κ*/*λ* ratio [26]. Results may suggest the intracerebral segregation of IgG-*κ* producing B cells and potential prognostic use in prediction of MS and conversion of CIS to DSMS [26, 29].

In November 2018, there was only one research carried out in Latin America. Sáez et al. proposed free light chain determination in CSF as an alternative for immunoglobulin G oligoclonal bands which showed that evaluation is difficult due to technical issues in some regions. The results were similar to those in other studies focused on multiple sclerosis; i.e., in those studies, high diagnostic sensitivity and specificity of *κ*FLCs for the diagnosis of MS were described. The authors also focused on the *λ*FLCs which had lower diagnostic sensitivity and specificity than *κ*FLCs. All patients had at least one brain MRI within the first 30 days of the study. The concentrations of free light chains tended to increase brain atrophy during follow-up. A study revealed strong correlation between the *κ*FLCs, *λ*FLCs, and the percentage brain volume change (PBVC) and an inverse correlation between *κ*/*λ* FLCs ratio and PBVC. Additionally, the authors compared FLC levels at CIS converters and CIS nonconverters that were similar in both conditions [27], while Voortman et al. found that the lower *κ*FLCs-*λ*FLCs ratio was associated with the risk of conversion to CDMS [24].

It is also worth considering a 2018 study about new biomarkers of pediatric multiple sclerosis including CSF free light chains. Ganelin-Cohen et al. showed similar results in adult population. The authors revealed abnormally elevated levels of *κ*FLCs, *λ*FLCs, and both *κ* and *λ* FLCs in the CSF of, respectively, 48%, 29%, and 14% of pediatric multiple sclerosis patients. Interestingly, “mixed” and *λ*FLCs cases were associated with more aggressive disease. In total, 90.5% of patients showed increased levels of *κ*FLCs monomers and dimers and/or increased levels of *λ* dimers. In summary, in this study, FLCs analysis showed higher diagnostic sensitivity and specificity for discrimination between MS and non-MS pediatric patients than using the oligoclonality test [43].

It should also be pointed out that in most studies, the control group consisted of patients with, e.g., noninflammatory CNS diseases, cranial/peripheral palsy, headaches, sensory disturbances, psychosomatic disorders, vertigo, neurodegeneration diseases, paralysis, Guillian-Barre syndrome, spinal diseases, and tumors. There was a highly elevated *κ*FLCs concentration and *κ*/*λ* CSF and usually moderately elevated *λ*FLCs in CIS/MS groups compared to controls. Other diseases associated with intrathecal infection such as neuroborreliosis showed moderate increases of both *κ*FLCs and *λ*FLCs levels in CSF [17]. Nevertheless, there is one study supporting diagnostic value of intrathecal FLC in neuroborreliosis patients. Hegen et al. showed that, despite a study in a small group, *κ*FLCs index and *λ*FLCs index were significantly elevated in neuroborreliosis than in controls. Therefore, it is suggested that the comparison of neuroborreliosis and multiple sclerosis needs to be performed [44].

For years, the diagnostic gold standard of intrathecal synthesis has been the detection of oligoclonal bands by isoelectric focusing and immunoblotting [45]. However, on the basis of literature data, we suggest that the *κ*FLCs index is the most interesting alternative for OCB analysis in diagnostics of MS which should be calculated together with OCB as a part of routine MS diagnosis. *κ*FLCs determination or *κ*FLCs index calculation can provide a rapid information about intrathecal inflammation.

## 3. HIV

Several infections of the central nervous system, including HIV infection, cause abnormalities of the humoral immunity. In HIV infected patients, the immune system is disrupted. Human immunodeficiency virus causes immune dysfunction, particularly characterized by hypergammaglobulinemia and chronic T- and B-cell activation. B-cell activation leads to elevated immunoglobulin synthesis and finally to an excess of free light chains.

### 3.1. HIV Infection

The diagnostic significance of free light chains in HIV-positive patients has been presented in Table 1.

HIV infection is demonstrated by the presence of increased levels of HIV-specific immunoglobulins. As a result, CSF oligoclonal bands are present in patients with HIV, asymptomatic seropositive patients (ASPs), and acquired immunodeficiency syndrome (AIDS). Therefore, some authors tried to investigate the light chain composition of OCB in sera and CSF of HIV-infected patients. In the 90s, first studies on the occurrence of free light chains in cerebrospinal fluid and serum in HIV-1 infection were published [18, 31–33]. A frequent occurrence of increased *κ* and *λ*FLCs was found in CSF and sera of HIV-1 infected patients. An elevation of free light chains was probably caused by activation of CSF B cells. Furthermore, Grimaldi et al. observed significant elevation of serum and CSF mean IgG *κ*/*λ*FLCs ratios which were associated with IgG *κ* OCB. In contrast, other researchers found out too that CSF *λ*FLCs show high intensity. However, in these cases, monomers were less frequent than dimers [18, 31, 33]. Moreover, there were variable correlations with the serum or CSF dimeric or monomeric FLCs form, but not with both of them. In our view, higher intensity of CSF dimers in AIDS than that in asymptomatic HIV-1 infection may be result of their intrathecal production in advanced stages of disease when the humoral immune functions are more dysregulated than in early stages. It is also crucial to mention that FLCs can be transferred from sera through the impaired blood-brain barrier and a qualitative demonstration of FLCs in the CSF may be significant only in the absence of altered BBB function [31].

Furthermore, Bibas et al. published the first study examining the associations between polyclonal FLCs sum and HIV biomarkers. This study revealed that advanced age patients with higher viral load, lower CD4 cell count, and shorter time of undetectable viremia had higher sum of *κ* and *λ* FLCs. Furthermore, the authors highlight the fact that patients with HCV coinfection had significantly higher levels of *κ* and *λ* FLCs [46]. In our opinion, it is worth pointing out that it was the first study on quantifying polyclonal FLCs sum in HIV infection, which could be a good diagnostic biomarker allowing us to identify patients who are at risk of immune dysfunction and of unresolved inflammation.

### 3.2. HIV: Antiretroviral Treatment

Antiretroviral treatment (ART) reduces the risk of virus transmission and suppresses HIV replication leading to reversion of B-cell defect and abnormalities and elimination of most infected CD4+ cells. ART has greatly reduced the morbidity and mortality of HIV-positive patients. There are some studies evaluating association between FLCs abnormal concentration and antiretroviral treatment [34, 35]. It has been proven that patients taking ART had lower levels of *κ* and *λ* with the ratio not significantly affected by ART use [34, 35]. Additionally, Zemlin et al. observed that *κ*FLCs and *λ*FLCs values correlated positively with viral load and IgG and negatively with CD4+ counts and albumin concentration [34]. The ratio correlated only with IgG levels. Abdulai et al. focused also on the proportions of CD21 revealing a CD21 cells correlation with FLCs in HIV-native patients and negative correlation with CD4+ counts. Moreover, FLCs in HIV-native patients correlated with IgG1, but not with IgG4. The authors also suggest that in HIV-treated patients, IgG1 correlation with *κ*FLCs was presumably caused by higher serum levels of *κ* compared to *λ* [35]. These results suggest that FLCs determinations can be a good screening and prognostic marker in monitoring HIV disease severity and antiretroviral therapy [34, 35]. Summarizing the results of published studies concerning FLCs in antiretroviral therapy of HIV infection, we believe that easy measurements of free light chains concentration are a strong predictive biomarker of treatment in patients with HIV.

### 3.3. HIV-Related Lymphomas

The progressive weakening of the immune system may predispose HIV-infected patients to dementia, cardiovascular disease, and bone, liver and renal disorders. However, indisputably, it is well known that HIV infection is also, a risk factor for several B-cell and non-AIDS malignances, including Hodgkin and non-Hodgkin lymphoma [36, 37, 46, 47]. It should also be pointed out that HIV-associated lymphoma compared to lymphoma in HIV-negative patients is characterized by advanced diseases occurring more often, extranodal involvement, and being usually associated with Epstein-Barr virus [16]. A few studies have shown that biomarkers for aberrant B-cell activation are elevated in HIV-positive patients who develop lymphomas. Shepherd et al., Tittle et al., Vendrame et al., and Baptista et al. tried to examine relationship of B-cell activation demonstrated among others by extending serum FLCs concentration and the risk of lymphoma in HIV-positive people. All these works described that in HIV-infected individuals, levels of FLCs were associated with the risk of lymphomas [36–39]. Moreover, Tittle et al. observed that 70% of patients had polyclonal increase of FLCs including 8% with monoclonal elevations. Because of polyclonal nature of FLCs, Bibas et al. evaluated the sum of *κ*FLCs and *λ*FLCs which revealed that *κ* + *λ* FLCs were significantly higher in patients with lymphoma. Importantly, increased FLCs concentration was associated with higher risk of non-Hodgkin than Hodgkin lymphoma. Presumably, it may be due to different pathogenic mechanisms of the prolonged immunosuppression. At the same time, a significant reduction in the risk of lymphoma was found in HIV-negative patients with low FLCs [40]. Tittle et al. also compared FLCs levels in three major lymphoma subtypes: Burkitt, diffuse large B cell, and Hodgkin. In about 75–80% of all those patients, FLCs were overproduced with no significant differences between these histological lymphoma subtypes. It can indicate the role of B-cell proliferation and hypergammaglobulinemia in the pathogenesis of all these lymphomas. They also concluded that there was no relationship between elevated free light chains concentration and overall survival. On the other hand, Shepherd et al. and Landgren et al. observed that *κ*FLCs and *λ*FLCs levels were moderately associated with lymphoma development amongst people with HIV (>2 years; 2–5 years, respectively) prior to lymphoma development. Accordingly, Vendrame et al. collected serum samples for three time points prior to non-Hodgkin lymphoma diagnosis. Serum *λ*FLCs levels were considered to be elevated at all three visits and *κ*FLCs at 1–3- and 3–5-year period. Landgren et al. also found that only *λ*FLCs was associated with non-Hodgkin lymphoma 0–2 years prior to diagnosis [36, 38, 41]. In contrast, levels of total IgG, IgM, and IgA were not associated with non-Hodgkin lymphoma. The signs seem to indicate that elevated FLCs levels may be a more-sensitive indicator of generalized disruption of B-cell functions. Furthermore, both Landgren et al. and Vendreme et al. observed that FLCs varied depending on CD4 count, and in the CD4 patients, *κ*FLCs and *λ*FLCs levels were linked to HIV- and AIDS non-Hodgkin lymphoma [38, 41]. Contrary to these findings, Bibas et al. found that FLCs levels have a good positive predictive value of non-Hodgkin and Hodgkin lymphomas, independent of CD4 cell count [40]. Interestingly, Babtista et al. tried to determine the diagnostic usefulness of FLCs and Epstein-Barr load combination of HIV-related Hodgkin and non-Hodgkin lymphomas. In parallel, direct polyclonal activation of B lymphocytes and the influence of Epstein-Barr virus infection are likely to have significant roles in the pathogenesis of HIV-associated lymphomas. They chose Epstein-Barr load, because it had been proven that Epstein-Barr virus infects and activates B cells with the load being increased in about 50% HIV-related lymphomas [38, 39]. It is well known that after infection with Epstein-Barr virus, human B lymphocytes actively secrete immunoglobulins. That is why, scientists observed higher FLCs concentrations in HIV-related lymphoma patients affected by the Epstein-Barr virus. They also described that Epstein-Barr loads were higher in patients with increased FLCs than in those with normal FLCs concentrations.

In summary, these results suggest that HIV-related immune system dysfunction is associated with elevated concentration of serum FLCs. FLCs are also a potentially strong and sensitive predictor of the risk of developing HIV-associated lymphomas.

## 4. Conclusion

Based on these published findings, we suggest that free light chains have high diagnostic sensitivity, which probably enables an application in laboratory diagnostics.

## Figures and Tables

**Table 1:** Significance of serum- and CSF-free light chains as candidates for markers of multiple sclerosis and HIV.

	Results	References
Multiple sclerosis	*κ*FLCs concentrations and *κ*FLCs index are highly elevated in MS compared to healthy and other CNS diseases	[17, 23–27]
	*λ*FLCs concentrations are moderately elevated in MS	[17, 27–29]
	*κ*FLCs concentrations are similar in MS and CIS	[17]
	*κ*FLCs index is higher in MS patients than in CIS patients	[23]
	*κ*FLCs-index has similar diagnostic sensitivity and specificity in comparison with OCB	[17, 25, 27, 30]
	*κ*FLCs index is lower in patients with nonactive disease	[24]
	*κ*FLCs levels are associated with IgG intrathecal synthesis	[17, 23, 29]
	*κ*FLCs index above cutoff point increases the risk of conversion CIS to MS	[23, 25]
	*κ*/*λ* ratio may predict conversion of CIS to MS	[24, 26, 27]

HIV	Serum and CSF *κ* and *λ* FLCs are increased in HIV-1 infected patients	[18, 31–33]
	*Κ* and *λ* FLCs are increased in ART-native patients compared to ART	[34, 35]
	*κ* and *λ* FLCs in ART patients correlate with IgG and viral load	[34]
	*κ* and *λ* FLCs in ART patients correlate negatively with CD4 and albumin	[34]
	In ART-native patients, FLCs correlate with CD21 and IgG	[35]
	*κ* and *λ* FLCs are elevated in HIV + patients who went on to develop lymphoma	[36–41]
	*κ* and *λ* FLCs correlate with Epstein-Barr load	[39]
	*κ* and *λ* FLCs are similar in different lymphoma subtypes	[37]
